# Machine learning based early warning system enables accurate mortality risk prediction for COVID-19

**DOI:** 10.1038/s41467-020-18684-2

**Published:** 2020-10-06

**Authors:** Yue Gao, Guang-Yao Cai, Wei Fang, Hua-Yi Li, Si-Yuan Wang, Lingxi Chen, Yang Yu, Dan Liu, Sen Xu, Peng-Fei Cui, Shao-Qing Zeng, Xin-Xia Feng, Rui-Di Yu, Ya Wang, Yuan Yuan, Xiao-Fei Jiao, Jian-Hua Chi, Jia-Hao Liu, Ru-Yuan Li, Xu Zheng, Chun-Yan Song, Ning Jin, Wen-Jian Gong, Xing-Yu Liu, Lei Huang, Xun Tian, Lin Li, Hui Xing, Ding Ma, Chun-Rui Li, Fei Ye, Qing-Lei Gao

**Affiliations:** 1 National Medical Center for Major Public Health Events, Tongji Hospital, Tongji Medical College, Huazhong University of Science and Technology, Wuhan, 430000 China; 2 Department of Gynecology and Obstetrics, Tongji Hospital, Tongji Medical College, Huazhong University of Science and Technology, Wuhan, 430000 China; 3 GNSS Research Center, Wuhan University, Wuhan, 430079 China; 4 City University of Hong Kong Shenzhen Research Institute, Shenzhen, 518000 China; 5 Department of Gastroenterology, Tongji Hospital, Tongji Medical College, Huazhong University of Science and Technology, Wuhan, 430000 China; 6 Department of Obstetrics and Gynecology, The Central Hospital of Wuhan, Tongji Medical College, Huazhong University of Science and Technology, Wuhan, China; 7 Department of Obstetrics and Gynecology, Xiangyang Central Hospital, Affiliated Hospital of Hubei University of Arts and Science, Xiangyang, Hubei China; 8 Department of Hematology, Tongji Hospital, Tongji Medical College, Huazhong University of Science and Technology, Wuhan, 430000 China; 9 Department of Neurosurgery, Tongji Hospital, Tongji Medical College, Huazhong University of Science and Technology, Wuhan, 430000 China

**Keywords:** Machine learning, Prognostic markers, Viral infection, Risk factors

## Abstract

Soaring cases of coronavirus disease (COVID-19) are pummeling the global health system. Overwhelmed health facilities have endeavored to mitigate the pandemic, but mortality of COVID-19 continues to increase. Here, we present a mortality risk prediction model for COVID-19 (MRPMC) that uses patients’ clinical data on admission to stratify patients by mortality risk, which enables prediction of physiological deterioration and death up to 20 days in advance. This ensemble model is built using four machine learning methods including Logistic Regression, Support Vector Machine, Gradient Boosted Decision Tree, and Neural Network. We validate MRPMC in an internal validation cohort and two external validation cohorts, where it achieves an AUC of 0.9621 (95% CI: 0.9464–0.9778), 0.9760 (0.9613–0.9906), and 0.9246 (0.8763–0.9729), respectively. This model enables expeditious and accurate mortality risk stratification of patients with COVID-19, and potentially facilitates more responsive health systems that are conducive to high risk COVID-19 patients.

## Abstract (web summary)

Methods to stratify patients according to mortality risk are essential to allocate limited heath resources during the COVID-19 crisis. Here, using machine learning methods, the authors present a mortality risk prediction model for COVID-19 that uses patients’ clinical data on admission to stratify patients by mortality risk.

## Introduction

Management of the surging infections of the coronavirus disease (COVID-19) is a huge clinical challenge. Currently, the pandemic is pummeling the global health system, with 18,902,735 people infected as of August 7, 2020^1–3^. The overwhelmed health facilities are unable to curb the increasing mortality of COVID-19^3^. Moreover, without proven effective treatments to date, patients who rapidly deteriorate into a refractory state harbor significantly higher risks of death^4,5^. Third, advanced COVID-19 is characterized by heterogeneous clinical features and multiorgan damage^5,6^, which requires an effective triage and intensive monitoring. Therefore, an early warning system that enables stratification of COVID-19 patients by risk of death on admission holds enormous promise to assist in the management of COVID-19.

Electronic health records (EHRs) abound with valuable information generated from routine clinical practices^7,8^, which can be useful for mortality risk prediction of COVID-19. However, data in EHRs are complex, multidimensional, nonlinear, and heterogeneous. Using models more effective than traditional statistical methods (univariate or multivariate Cox regressions and logistic regression (LR)) for analysis can help to fully utilize the clinical data in EHRs. Machine learning (ML), a subfield of artificial intelligence, encapsulates statistical and mathematical algorithms that enable facts interrogation and complex decision-making^9,10^. Therefore, combinatory uses of ML algorithms and EHRs for prognosis prediction in the context of COVID-19 pandemic are worth exploring.

ML algorithms have been explored in myriad fields of COVID-19 including, but not limited to, detecting outbreaks, identification and classification of COVID-19 medical images, rapid diagnosis, severity risk prediction, and prognosis prediction^11–15^. For COVID-19 patients and clinicians, the greatest concern is whether the patients can survive. Available ML models that focus on this exhibit promising prognostic implications, but are still impeded by the paucity of external validations and limited follow-ups, and lack the capability of predicting prognosis as early as the time of admission.

In this study, we aim to develop a mortality risk prediction model for COVID-19 (MRPMC) that utilizes clinical data in EHRs to stratify patients by mortality risk on admission. The validated capability of enabling expeditious and accurate mortality risk stratification of COVID-19 may facilitate more responsive health systems that are conducive to high-risk COVID-19 patients via early identification, and ensuing instant intervention as well as intensive care and monitoring, thus, hopefully assisting to save lives during the pandemic.

## Results

### Study design and baseline characteristics

**Fig. 1:**
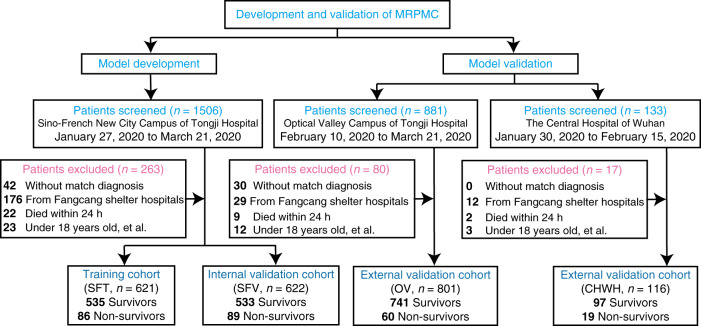
Study design. MRPMC mortality risk prediction model for COVID-19, SFT training cohort of Sino-French New City Campus of Tongji Hospital, SFV internal validation cohort of Sino-French New City Campus of Tongji Hospital, OV Optical Valley Campus of Tongji Hospital, CHWH The Central Hospital of Wuhan.

To train and validate the MRPMC for prognosis prediction of COVID-19, we included 2520 consecutive COVID-19 patients with known outcomes (discharge or death) from two affiliated hospitals of Tongji Medical College, Huazhong University of Science and Technology, including Sino-French New City Campus of Tongji Hospital (SF) and Optical Valley Campus of Tongji Hospital (OV), and The Central Hospital of Wuhan (CHWH) between January 27, 2020 and March 21, 2020. As a total of 360 patients were excluded with definite reasons, 2160 COVID-19 patients met eligibilities. For detailed exclusions, see Fig. 1 and “Methods,” participants. We randomly partitioned 50 and 50% of participants from SF into the training cohort (SFT cohort) and internal validation cohort (SFV cohort), respectively. Participants from OV and CHWH were used as two external validation cohorts (OV cohort and CHWH cohort). Compositions of the four cohorts are displayed in Fig. 1 and “Methods,” cohorts. The study design has been schematically presented in Fig. 1 and Supplementary Fig. 1.

Table 1 shows the baseline characteristics of the four cohorts. The median age of the participants was 62 years (interquartile range [IQR]: 51–71) in the SFT cohort, 63 years (IQR: 51–70) in the SFV cohort, 63 years (IQR: 50–70) in the OV cohort, and 62.5 years (IQR: 55–72) in the CHWH cohort. The male patients accounted for 50.7, 50.0, 46.7, and 54.3% of all participants in the SFT, SFV, OV, and CHWH cohorts, respectively. Hypertension (37.1–40.3%) was the most prevalent comorbidity and fever (61.2–86.0%) remained the most common symptom. The median time from admission to death or discharge ranged from 17 to 23 days among all four cohorts.

**Table 1:** Baseline characteristics of individuals by cohort.

	SFT cohort	SFV cohort	OV cohort	CHWH cohort
Characteristics	(*n* = 621)	(*n* = 622)	(*n* = 801)	(*n* = 116)
Age	62 (51–71)	63 (51–70)	63 (50–70)	62.5 (55–72)
Sex				
Female	306 (49.3%)	311 (50.0%)	427 (53.3%)	53 (45.7%)
Male	315 (50.7%)	311 (50.0%)	374 (46.7%)	63 (54.3%)
Comorbidity number	1 (0–2)	1 (0–2)	1 (0–2)	2 (1–3)
Comorbidity				
Hypertension	245 (39.5%)	244 (39.2%)	321 (40.3%)	43 (37.1%)
Diabetes	110 (17.7%)	110 (17.7%)	121 (15.2%)	16 (13.8%)
CHD	72 (11.6%)	59 (9.5%)	68 (8.5%)	16 (13.8%)
CLD	26 (4.2%)	19 (3.1%)	33 (4.1%)	7 (6.0%)
Tumor	22 (3.5%)	21 (3.4%)	20 (2.5%)	51 (44.0%)
HBV	16 (2.6%)	13 (2.1%)	24 (3.0%)	1 (1.0%)
CKD	13 (2.1%)	8 (1.3%)	11 (1.4%)	1 (0.9%)
COPD	4 (0.6%)	7 (1.1%)	7 (0.9%)	1 (0.9%)
Fever	533 (86.0%)	527 (84.9%)	584 (73.0%)	71 (61.2%)
Temp (max) ≥ 39 °C	169 (27.4%)	194 (31.5%)	158 (19.8%)	16 (14.2%)
Cough	450 (72.6%)	436 (70.2%)	601 (75.1%)	63 (54.3%)
Dyspnea	313 (50.5%)	283 (45.6%)	274 (34.2%)	37 (31.9%)
Sputum	233 (37.6%)	228 (36.7%)	344 (43.0%)	32 (27.6%)
Fatigue	253 (40.8%)	233 (37.5%)	250 (31.2%)	43 (37.1%)
Diarrhea	186 (30.0%)	167 (26.9%)	135 (16.9%)	9 (7.8%)
Myalgia	133 (21.5%)	144 (23.2%)	129 (16.1%)	20 (17.2%)
Vomiting	30 (4.8%)	31 (5.0%)	32 (4.0%)	3 (2.6%)
Conscious at admission	595 (95.8%)	600 (96.5%)	786 (98.1%)	79 (68.1%)
Respiratory rate, per min	20 (20–22)	21 (20–24)	21 (20–24)	21 (20–24)
MAP, mmHg	96.7 (88.7–104.7)	97.2 (89.7–105.6)	96.3 (87.7–106.7)	93.3 (86.9–101.5)
SpO_2_, %	95 (91–97)	95 (91–97)	96 (94–97)	95.5 (93–97.3)
Vital status				
Death	86 (13.8%)	89 (14.3%)	60 (7.5%)	19 (16.4%)
Discharge	535 (86.2%)	533 (85.7%)	741 (92.5%)	97 (83.6%)
Follow-up, days	23 (15–30)	21 (15–29)	19 (14–26)	17 (12–24)

Continuous variables are presented as median (interquartile ranges [IQR]), while categorical variables as counts and percentages (%).

*SFT cohort* training cohort of Sino-French New City Campus of Tongji Hospital, *SFV cohort* internal validation cohort of Sino-French New City Campus of Tongji Hospital, *OV cohort* external validation cohort of Optical Valley Campus of Tongji Hospital, *CHWH cohort* external validation cohort of The Central Hospital of Wuhan, *Follow-up* time from admission to death or discharge, *CHD* coronary heart disease, *CLD* chronic liver disease, *HBV* hepatitis B virus, *CKD* chronic kidney disease, *COPD* chronic obstructive pulmonary disease, *MAP* mean arterial pressure.

### Features selected by least absolute shrinkage and selection operator (LASSO)

**Fig. 2:**
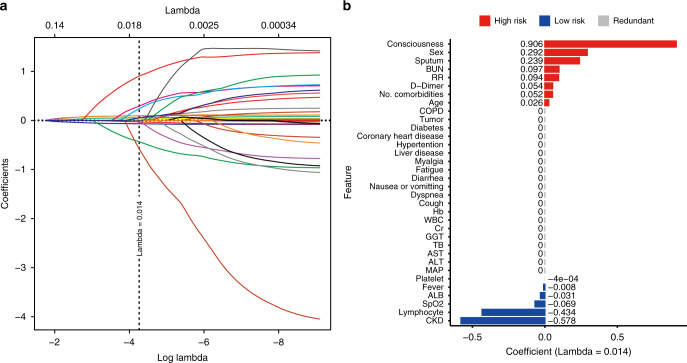
Feature selection by LASSO. **a** LASSO variable trace profiles of the 34 features whose intracohort missing rates were less than 5%. The vertical dashed line shows the best lambda value 0.014 chosen by tenfold cross validation. **b** Feature coefficient of LASSO with best lambda value 0.014. High-risk (positive coefficient) and low-risk (negative coefficient) features are colored in red and blue, respectively. Gray features with coefficient 0 were considered redundant and removed, resulting in 14 features left for downstream prognosis modeling. LASSO least absolute shrinkage and selection operator, BUN blood urea nitrogen, RR respiratory rate, COPD chronic obstructive pulmonary disease, Hb hemoglobin, WB, white blood cell count, Cr creatinine, GGT gamma-glutamyl transferase, TB total bilirubin, AST aspartate aminotransferase, ALT alanine transaminase, MAP mean arterial pressure, ALB albumin, SpO_2_ oxygen saturation, CKD chronic kidney disease.

Among 53 raw features extracted from EHRs (Supplementary Table 1), those with a proportion of missing values greater than or equal to 5% in each cohort were filtered (Supplementary Fig. 2), resulting in 34 features, including 18 categorical features and 16 continuous ones (Supplementary Fig. 3 and 4) that underwent feature selection by the LASSO (Fig. 2a). Only 14 of the 34 features were eventually chosen for modeling (Fig. 2b), among which 8 features had a positive association with mortality (high risk: consciousness, male sex, sputum, blood urea nitrogen [BUN], respiratory rate [RR], D—dimer, number of comorbidities, and age) and 6 features were negatively correlated with mortality (low risk: platelet count [PLT], fever, albumin [ALB], SpO_2_, lymphocyte, and chronic kidney disease [CKD]). Multivariable Cox analysis using raw data of the 34 features proved that the features selected by LASSO exhibited similar prognostic implications (Supplementary Fig. 5 and Supplementary Table 2). High-risk features identified by LASSO were also significant unfavorable prognostic indicators recognized via multivariable Cox analysis (hazard ratio [HR] > 1 and *p* < 0.05). Similarly, low-risk features accorded with favorable prognostic indicators (HR < 1 and *p* < 0.05).

### Model performance

**Fig. 3:**
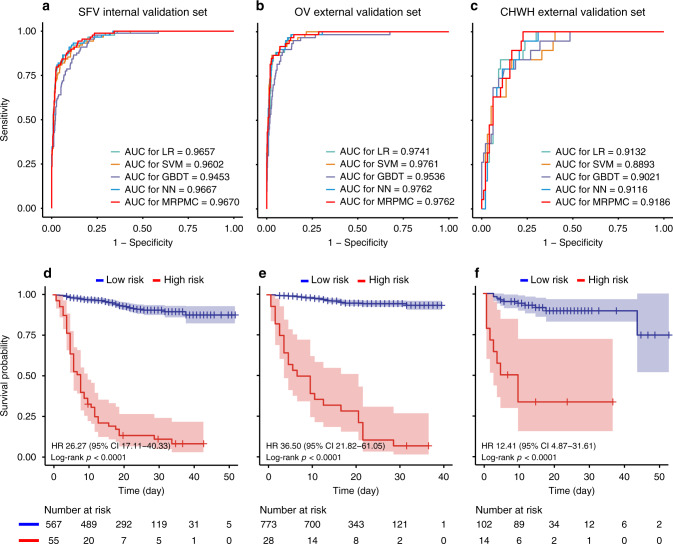
Predictive performance of models across cohorts. AUC to assess the performance of mortality risk prediction of models (LR, SVM, GBDT, NN, and MRPMC) in **a** SFV cohort, **b** OV cohort, and **c** CHWH cohort, respectively. Source data are provided as a Source Data file. Kaplan–Meier curves indicating overall survival of patients with high and low mortality risk in **d** SFV cohort, **e** OV cohort, and **f** CHWH cohort, respectively. The tick marks refer to censored patients. The dark red or blue line indicates the survival probability, and the light red or blue areas represent the 95% confidence interval of survival probability (*p* < 0.0001). *AUC* area under the receiver operating characteristics curve, *SFV* internal validation cohort of Sino-French New City Campus of Tongji Hospital, *OV* Optical Valley Campus of Tongji Hospital, *CHWH* The Central Hospital of Wuhan, *LR* logistic regression, *SVM* support vector machine, GBDT gradient boosted decision tree, NN neural network, MRPMC mortality risk prediction model for COVID-19.

In general, six ML models including LR, support vector machine (SVM), gradient boosted decision tree (GBDT), neural network (NN), K-nearest neighbor (KNN), and random forest (RF) all displayed varying but promising performances to predict mortality risk in the three validation cohorts in terms of discrimination and calibration. To build a predictive model with augmented prognostic implications, we integrated the top four best predictive models (LR, SVM, GBDT, and NN) to create an ensemble model called MRPMC. MRPMC outputted a normalized probability of mortality risk ranging from 0 to 1. We selected the threshold of 0.6 to assign the predicted mortality risk label by optimizing F1 score on the training cohort (Supplementary Fig. 6). Probabilities of less than 0.6 were assigned to low risk and otherwise to high risk for all ML methods across all cohorts. The procedures of establishing the MRPMC are elaborated in Methods, Model development. As expected, MRPMC exhibited greater capability of predicting mortality risk of COVID-19 than the four contributive models alone in the SFV and CHWH cohorts, though the differences between SVM and MRPMC were nuanced in the OV cohort (Fig. 3a–c).

MRPMC achieved an area under the receiver operating characteristics (ROC) curve (AUC) of 0.9621 (95% confidence interval [CI]: 0.9464–0.9778) in identification of nonsurvivors with an accuracy of 92.4% (95% CI: 90.1–94.4%) in SFV cohort. For OV cohort, MRPMC demonstrated an AUC of 0.9760 (95% CI: 0.9613–0.9906) and an accuracy of 95.5% (95% CI: 93.8–96.8%) to predict prognosis of COVID-19. An AUC of 0.9246 (95% CI: 0.8763–0.9729) and an accuracy of 87.9% (95% CI: 80.6–93.2%) for prognosis prediction were observed for CHWH cohort (Table 2). The calibration curve of MRPMC in the three validation cohorts are depicted in Supplementary Fig. 7, showing that MRPMC displayed a Brier score of 0.051 for SFV cohort, 0.029 for OV cohort, and 0.083 for CHWH cohort. Performances of four contributing algorithms are listed in Table 2, and that of the other two ML models (KNN and RF) in Supplementary Fig. 8 and Supplementary Table 3.

**Table 2:** Performance for mortality risk prediction of models in validation cohorts.

	AUC (95% CI)	Accuracy (95% CI)	Sensitivity (95% CI)	Specificity (95% CI)	PPV (95% CI)	NPV (95% CI)	F1	Kappa	Brier
Internal validation cohort (SFV)									
MRPMC	0.9621 (0.9464–0.9778)	92.4% (90.1–94.4%)	57.3% (46.4–67.7%)	98.3% (96.8–99.2%)	85.0% (73.4–92.9%)	93.2% (90.8–95.2%)	0.685	0.644	0.051
SVM	0.9594 (0.9424–0.9764)	92.4% (90.1–94.4%)	60.7% (49.8–70.9%)	97.8% (96.1–98.8%)	81.8% (70.4–90.2%)	93.7% (91.4–95.6%)	0.697	0.655	0.052
GBDT	0.9454 (0.9246–0.9662)	91.5% (89.0–93.6%)	60.7% (49.8–70.9%)	96.6% (94.7–98.0%)	75.0% (63.4–84.5%)	93.6% (91.3–95.5%)	0.696	0.643	0.066
LR	0.9614 (0.9456–0.9772)	92.1% (89.7–94.1%)	56.2% (45.3–66.7%)	98.1% (96.6–99.1%)	83.3% (71.5–91.7%)	93.1% (90.6–95.0%)	0.671	0.628	0.051
NN	0.9615 (0.9456–0.9774)	92.1% (89.7–94.1%)	51.7% (40.8–62.4%)	98.9% (97.6–99.6%)	88.5% (76.6–95.7%)	92.5% (90.0–94.5%)	0.653	0.612	0.051
External validation cohort (OV)									
MRPMC	0.9760 (0.9613–0.9906)	95.5% (93.8–96.8%)	45.0% (32.1–58.4%)	99.6% (98.8–99.9%)	90.0% (73.5–97.9%)	95.7% (94.0–97.0%)	0.600	0.579	0.029
SVM	0.9774 (0.9640–0.9908)	95.8% (94.1–97.0%)	50.0% (36.8–63.2%)	99.5% (98.6–99.9%)	88.2% (72.6–96.7%)	96.1% (94.5–97.4%)	0.638	0.618	0.028
GBDT	0.9536 (0.9279–0.9793)	94.8% (93.0–96.2%)	48.3% (35.2–61.6%)	98.5% (97.4–99.3%)	72.5% (56.1–85.4%)	95.9% (94.3–97.2%)	0.580	0.553	0.039
LR	0.9721 (0.9568–0.9875)	95.4% (93.7–96.7%)	45.0% (32.1–58.4%)	99.5% (98.6–99.9%)	87.1% (70.2–96.4%)	95.7% (94.0–97.0%)	0.593	0.572	0.031
NN	0.9754 (0.9602–0.9906)	95.6% (94.0–96.9%)	46.7% (33.7–60.0%)	99.6% (98.8–99.9%)	90.3% (74.3–98.0%)	95.8% (94.2–97.1%)	0.615	0.595	0.028
External validation cohort (CHWH)									
MRPMC	0.9246 (0.8763–0.9729)	87.9% (80.6–93.2%)	42.1% (20.3–66.5%)	96.9% (91.2–99.4%)	72.7% (39.0–94.0%)	89.5% (82.0–94.7%)	0.533	0.470	0.083
SVM	0.9067 (0.8482–0.9652)	88.8% (81.6–93.9%)	57.9% (33.5–79.8%)	94.6% (88.4–98.3%)	68.8% (41.3–89.0%)	92.0% (84.8–96.5%)	0.629	0.563	0.090
GBDT	0.9021 (0.8347–0.9694)	87.9% (80.6–93.2%)	31.6% (12.6–56.6%)	99.0% (94.4–100.0%)	85.7% (42.1–99.6%)	88.1% (80.5–93.5%)	0.462	0.410	0.089
LR	0.9213 (0.8710–0.9717)	87.1% (79.6–92.6%)	36.8% (16.3–61.6%)	96.9% (91.2–99.4%)	70.0% (34.8–93.3%)	88.7% (81.1–94.0%)	0.483	0.417	0.091
NN	0.9202 (0.8700–0.9705)	88.8% (81.6–93.9%)	47.4% (24.5–71.1%)	96.9% (91.2–99.4%)	75.0% (42.8–94.5%)	90.4% (83.0–95.3%)	0.581	0.520	0.083

*SFV* internal validation cohort of Sino-French New City Campus of Tongji Hospital, *OV* Optical Valley Campus of Tongji Hospital, *CHWH* The Central Hospital of Wuhan, *MRPMC* mortality risk prediction model for COVID-19, *SVM* support vector machine, *GBDT* gradient boosted decision tree, *LR* logistic regression, *NN* neural network, *AUC* area under the receiver operating characteristics curve, *PPV* positive predictive value, *NPV* negative predictive value, *95% CI* 95% confidence interval.

Moreover, with the time from admission to death or discharge as the endpoint, Kaplan–Meier analysis further confirmed that MRPMC could robustly stratify patients by mortality risk. High-risk COVID-19 patients labeled by MRPMC were significantly less likely to survive than low-risk patients in the SFV, OV, and CHWH validation cohorts (Fig. 3d–f; *p* < 0.0001) with an HR of 26.85 (95% CI: 17.41–41.42), 32.83 (95% CI: 19.70–54.70), and 12.81 (95% CI: 5.09–32.24), respectively, highlighting the capability of MRPMC to accurately predict prognosis of COVID-19.

### Analyzing features included in models

**Fig. 4:**
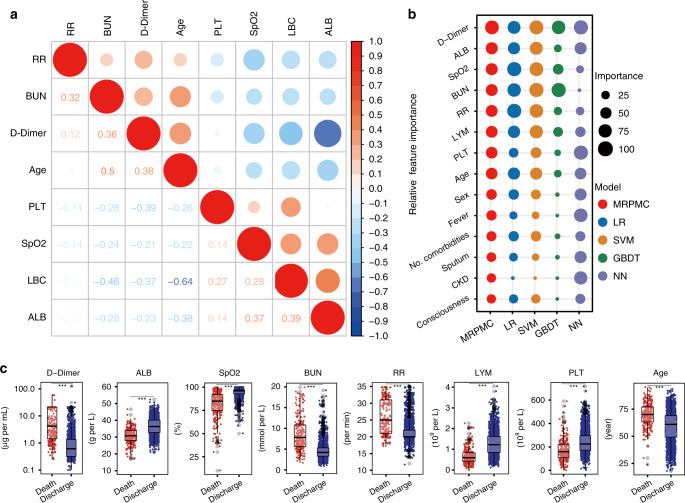
Statistical analysis of features included in models. **a** Heatmap representing the correlation between continuous features included in MRPMC using Spearman’s correlation coefficient. The colors in the plot represent the correlation coefficients. The redder the color, the stronger the positive monotonic relationship. The bluer the color, the stronger the negative monotonic relationship. The size of the circle represents the absolute value of the correlation coefficient, where a larger circle represents a stronger correlation. The numbers in the lower triangle represent the value of correlation coefficient. **b** Scaled importance rank of all features included in MRPMC for identifying high mortality risk COVID-19 patients included in the models. The size of circles represents the value of relative importance. The different color of circles represents the feature importance in different models. **c** Box and jitter plots showing distribution of continuous features included in MRPMC between deceased patients (*n* = 254) and discharged patients (*n* = 1906). The center line represents the median of the feature. Box limits represent upper and lower quartiles. Whiskers represent 1.5 times interquartile range. Gray points represent outliers. The median [IQR] of the features shown in Fig. 4c were listed in Supplementary Table 4. Wilcoxon test was used in the univariate comparison between groups and a two-tailed *p* < 0.05 was considered as statistically significant. ***p < 0.001. Source data are provided as a Source Data file. *MRPMC* mortality risk prediction model for COVID-19, *ALB* albumin, *SpO*_*2*_ oxygen saturation, *BUN* blood urea nitrogen, *RR* respiratory rate, *LYM* lymphocyte count, *PLT* platelet count, *No. comorbidities* number of comorbidities, *CKD* chronic kidney disease, *IQR* interquartile range.

Eight continuous features included in MRPMC exhibited correlation to varying degrees (Fig. 4a). Relative importance rank of all 14 variables for mortality prediction in MRPMC and the four contributive models are illustrated in Fig. 4b and Supplementary Table 4. The top weighted features (elevated D-dimer, decreased SpO_2_, increased RR, and lymphocytopenia) coincided with previously reported risk factors that were highly correlated with poor outcome in COVID-19^4,5^. Standard box plots presented all differential continuous variables between survivors and nonsurvivors (Fig. 4c). Nonsurvivors had significantly (*p* < 0.001) advanced age, higher levels of BUN and D-dimer, and lower levels of SpO_2_, lymphocyte, ALB, and PLT (Fig. 4c and Supplementary Table 5). These findings were also parallel to risk factors of mortality of COVID-19 delineated previously^16^, indicating that the selected features were highly relevant to prognosis.

## Discussion

In this multicenter retrospective study, we built the MRPMC, an ensemble model derived from four ML algorithms (LR, SVM, GBDT, and NN), that enabled accurate prediction of physiological deterioration and death for COVID-19 patients up to 20 days in advance using clinical information in EHRs on admission, and validated it both internally and externally. Importantly, the MRPMC displayed an AUC ranging from 0.9186 to 0.9762 in the three validation cohorts. The prognostic implications of MRPMC might facilitate more responsive health systems that are conducive to high-risk COVID-19 patients via early identification, and ensuing instant intervention as well as intensive care and monitoring, thus, hopefully assisting to save lives during the pandemic.

Generalizability was the first advantage of MRPMC. Initially, the SFV and OV cohorts comprised patients from two designated campuses for COVID-19, where 40 top-level medical teams across China collaborated to eradicate the crisis. Patients in the CHWH cohort were treated in a general hospital. Therefore, medical records on admission were more comprehensive in SFV and OV cohorts than in CHWH, and the treatments that patients received throughout hospitalization were more parallel between SFV and OV cohorts. Second, 44% of participants in CHWH cohort were COVID-19 patients with malignancy who were more vulnerable to COVID-19 and less likely to survive than noncancerous COVID-19 patients^17,18^. Validation of MRPMC in the CHWH cohort offered us opportunities not only to predict mortality risk in COVID-19 patients with cancer, a group where prognosis prediction is particularly pivotal and challenging, but also to assess MRPMC in an external validation cohort with heterogenous baseline characteristics. Importantly, although the settings of the validation cohorts varied, MRPMC exhibited an AUC of 0.9186 (95% CI: 0.8686–0.9687) to identify high-risk patients in the CHWH cohort, indicating that the prognostic implications of MRPMC were not confined to cohorts similar to SFT, but could also be successfully validated in an inhomogeneous cohort.

Strengths of MRPMC also include its stability and practicability upon COVID-19 patients with several missing features. To begin with, the 14 features for prognosis prediction were readily accessible and frequently monitored in routine clinical practice. Age and sex were basic information. Fever, sputum, and consciousness were easily observed symptoms, while RR and SpO_2_ were physical signs available at hand. Presence of CKD and number of comorbidities could be ascertained by referring to previous EHRs and patients or their family doctors. PLT, BUN, D-dimer, ALB, and lymphocytes were low-cost laboratory tests and conveniently determined. Unlike self-reported symptoms, these features were relatively more objective and solid, and less susceptible to memory bias. Though the 14 features were readily accessible, we appreciated the differences in medical procedures and uneven distribution of medical resources among different regions, countries, and continents. The missing features may thwart those who imminently need MRPMC. Importantly, with the imputation method we adopted (see Methods), MRPMC could still perform well in patients with several missing features.

In addition, MRPMC had certain interpretability. Features contributing to mortality risk prediction in this study were tangible and many of them had been proven intimately correlated with mortality in COVID-19 patients. Advanced age, male sex, and presence of multiple comorbidities were identified as risk factors associated with death in COVID-19 patients^4,5^. Sputum, supraphysiologic RR, and decreased SpO_2_ were directly related to pulmonary abnormalities in COVID-19. Elevated BUN, increased D-dimer, and lymphocytopenia might indicate extrapulmonary disorders and were potentially correlated with multiorgan damage caused by COVID-19^4,5^.

Available ML-based studies on prognosis prediction of COVID-19 patients are impeded by limited sample size, category of variables for prediction, short-term follow-ups for outcomes, and paucity of independent external validation^19–26^. To overcome these obstacles, we included 2520 consecutive inpatients with definite outcomes and detailed baseline characteristics within a specific time period for training and multiple validations of MRPMC to avoid overfitting and ensure general applicability, reproducibility, and credibility. Meanwhile, the features contributing to prognosis prediction were collected and proposed by a multidisciplinary team including experienced clinicians, epidemiologists, and informaticians, which guaranteed the representativeness of features. Importantly, time from admission to death or discharge was 21 (IQR: 15–29) days, 19 (IQR: 14–26) days, and 17 (IQR: 12–24) days in the SFV, OV, and CHWH validation cohorts, respectively. As MRPMC displayed impressive AUCs to predict mortality risk in the validation cohorts, it could predict death ~20 days in advance. Last, since the characteristics of datasets could affect the validity of the classification strategies of ML algorithms, we proposed an ensemble model derived from four ML algorithms for more accurate prediction of mortality risk in COVID-19 patients.

Although most cases of COVID-19 are not life-threatening, those that underwent physiological deterioration harbored significantly higher mortality (49.0% for critically ill patients versus 2.3% for overall patients)^27^. As the pandemic causes more infections, our understandings of the risk factors for mortality and the role that supportive, targeted, and immunological therapies play in treating COVID-19 continue to improve^16,28,29^. The aim of developing MRPMC is to mitigate the huge burden derived from COVID-19 on global health system and help to optimize clinical decision makings. MRPMC could automatically identify patients having high mortality risk as early as the time of admission when related symptoms are mild and nonspecific. This group of patients needs intensive monitoring and instant treatment when unfavorable prognostic indicators are observed, thus, hopefully improving patient outcomes. However, multiple evaluations of MRPMC in larger cohorts, prospective settings, and clinical trials are needed before elucidating its contribution to improving outcome of COVID-19^15^.

This study had some limitations. Patients included were primarily local residents from Wuhan, China. The predictive performance of the ML models merits investigation in other regions and ethnicities. Besides, the prognostic implications of MRPMC have not been evaluated in prospective cohorts due to the retrospective nature of this study.

In conclusion, combinatorial applications of MRPMC and EHRs with readily available features can enable timely and accurate risk stratification of COVID-19 patients on admission. MRPMC can potentially assist clinicians to promptly target the high-risk patients on admission, and accurately predict physiological deterioration and death up to 20 days in advance.

## Methods

### Participants

We included 2520 consecutive COVID-19 patients with known outcomes (discharge or death) from two affiliated hospitals of Tongji Medical College, Huazhong University of Science and Technology (Sino-French New City Campus of Tongji Hospital, SF and Optical Valley Campus of Tongji Hospital, OV) and The Central Hospital of Wuhan (CHWH) between January 27, 2020 and March 21, 2020. A total of 360 patients were excluded for various reasons, including 72 patients who failed to accord with the defined diagnosis of COVID-19 in the 7th edition of the Diagnosis and Treatment Protocol of COVID-19 released by the National Health Commission of China^30^, 217 patients who were transferred from Fangcang shelter hospitals for isolation, 33 patients who died within 24 h of admission, and 38 patients who were under 18 years of age, were pregnant, or were re-hospitalized or discharged for special reasons such as dialysis (Fig. 1). Eventually, 2160 patients were included for model training and validations.

### Cohorts

We randomly partitioned 50 and 50% of participants from SF into training cohort (SFT cohort) and internal validation cohort (SFV cohort), respectively. Participants from OV and CHWH were used as two external validation cohorts (OV cohort and CHWH cohort). Specifically, as Fig. 1 indicates, SFT cohort comprised 621 patients (535 survivors and 86 nonsurvivors); SFV, 622 patients (533 survivors and 89 nonsurvivors); OV, 801 patients (741 survivors and 60 nonsurvivors); and CHWH, 116 patients (97 survivors and 19 nonsurvivors). Patients with malignancy were reportedly more susceptible and vulnerable to COVID-19 owing to their immunocompromised states caused by the cancer itself, cachexia, and antitumor treatment^31^. They were also less likely to survive than noncancerous COVID-19 patients^17,18^, making COVID-19 patients with cancer an intriguing group of population for prognosis prediction. To investigate the capability of ML models to predict prognosis in this population, we consecutively included 54 malignant COVID-19 patients from the Cancer Center of CHWH and 62 noncancerous COVID-19 patients from the Department of Respiratory of CHWH to constitute another external validation cohort. The detailed baseline characteristics of the cohorts are shown in Table 1.

### Ethics

This study was approved by the Research Ethics Commission of Tongji Medical College, Huazhong University of Science and Technology (TJ-IRB20200406) with waived informed consent by the Ethics Commission mentioned above. This study was part of the observational clinical trial titled “A retrospective study for evolution and clinical outcomes study of novel coronavirus pneumonia (COVID-19) patients,” which was registered in the Chinese Clinical Trial Registry (ChiCTR2000032161). The clinical trial partly aimed to investigate the independent risk factors for adverse outcomes of COVID-19. The detailed information can be accessed in http://www.chictr.org.cn/showprojen.aspx?proj=52561.

### Data collection

Under the guidance of a multidisciplinary team including experienced clinicians, epidemiologists, and informaticians, we extracted 53 features including epidemiological, demographic, clinical, laboratory, radiological, and outcome data from EHRs using identical data collection forms on the first day of admission (Supplementary Table 1). Trained researchers entered and double-checked the data independently. To ensure the alarming function and subjective initiative of models, we abandoned variables generated in the late admission and variables regarding treatment. For patients with multiple features, we included only the first episode in various categories at admission.

### Feature filtering and imputation

First, we removed 19 features that harbored a proportion of missing values greater than or equal to 5% in each cohort (Supplementary Fig. 2). Filtering features with a large fraction of missing entries is common when dealing with clinical data^32^. Then, we imputed the missing entries with R-package missForest in the three cohorts separately^33^. Imputation of clinical data with RF has been widely adopted^34,35^, which displayed the capability of handling mix-type missing values including continuous and categorical variables. Although we visualized the imputation result of categorical and continuous variables separately (Supplementary Fig. 3 and 4), the imputation was conducted with the 34 mix-type features together for three validation cohort, respectively.

### Feature selection

After filtering 19 features and data imputation, there were 34 features remaining for feature selection. To eliminate redundant collinear features and diminish cost of clinical testing, we performed feature selection by recognizing the most predictive variables using LASSO LR (Fig. 2a)^32,36^. LASSO added the L1 norm of the feature coefficients as a penalty term to the loss function, which forced the coefficients corresponding to those weak features to become zero. Herein, we considered features whose coefficients were equal to zero as redundant features and abandoned them, resulting in 14 selected features for model constructions (Fig. 2b).

### Model development

We trained the models to predict mortality risk with the 14 variables and outcomes of COVID-19 patients. During model training, we fitted six baseline ML models, including LR, SVM, KNN, RF, GBDT, and NN, into the SFT cohort with tenfold cross validation to fine-tune the model parameters. Increasing the weight of minority categories in the model can increase the punishment for wrong classification of minority categories during training, and improve the model’s ability to recognize minority categories^37^. Therefore, we adopted weighted cross-entropy and increased the weight of class death for probability-based classifiers (LR, RF, GBDT, and NN). Subsequently, an ensemble model derived from four baseline models of best predictive performance (LR, SVM, GBDT, and NN), named MRPMC, was proposed by weighted voting. Specifically, the mortality risk probability of each individual estimator (LR, SVM, GBDT, and NN) was integrated by manually assigning weights with 0.25, 0.3, 0.1, and 0.35, respectively. After all ML models were well fitted, they were internally and externally evaluated in SFV, OV, and CHWH cohorts. Herein, we modeled the mortality prediction task as a binary classification problem. All included ML models output a normalized probability of mortality risk range from 0 to 1. We selected the threshold of 0.6 to assign the predicted mortality risk label by optimizing F1 score on the training cohort (Supplementary Fig. 6). Probabilities of less than 0.6 were assigned to low risk and otherwise to high risk for all ML methods across all cohorts. R library caret was utilized for model training and prediction. The LR, SVM, KNN, RF, GBDT, and NN models were called with method bayesglm, svmLinear, knn, rf, gbm, and avNNet with default settings, respectively. We standardized the features data with BoxCox, center, and scale function before training and prediction. Especially, we first adopted BoxCox transformation to make the data distribution more Gaussian-like^38^, and then standardized features by subtracting the mean and scaling to unit variance. Variable *z* was calculated as: $z = (x-u)/s$, where *u* was the mean and *s* was the standard deviation of the variable.

### Model evaluation

The predictive performance of the models was evaluated by ROC curve, Kaplan–Meier curve, calibration curve, and evaluation metrics including area under the ROC curve (AUC), accuracy, sensitivity, specificity, positive predictive value (PPV), negative predictive value (NPV), F1 score, Cohen’s Kappa coefficient (Kappa), and Brier score. The relative feature importance of each model was calculated using varImp function in caret R package. As SVM and KNN classifier had no built-in importance score, the AUC for each feature was utilized as the importance score.

### Statistical analysis

Statistical analysis was performed in R (version 3.6.2). For descriptive analysis, median (IQR) and frequencies (%) were assessed for continuous and categorical variables, respectively. The ROC curve and AUC analysis were conducted with R pROC package. Accuracy, sensitivity, specificity, PPV, NPV, Kappa, and F1 score were calculated with R caret and epiR packages. The calibration curve and Brier score were obtained with R-package rms. Relative feature importance was calculated using R-package caret. Survival curves were developed by Kaplan–Meier method with log-rank test, and plotted with R-package survival and survminer. Comparison of continuous variables was achieved by the Mann–Whitney *U* test using R-package table1. Odds ratio and corresponding 95% CI from LR were calculated with R-package stats. The significance level was set at a two-sided *p* value below 0.05. Univariate and multivariate Cox regression was utilized to calculate the HR with R-package survival. All dry-lab experiments were conducted in three different computing servers with consistent result.

### Reporting summary

Further information on research design is available in the Nature Research Reporting Summary linked to this article.

## Supplementary information

Supplementary Information

Reporting Summary

## Data Availability

Data pertaining to the patients’ features used for modeling are available to researchers upon reasonable request via contacting the corresponding author. Patient current vital status and follow-up information are not publically available due to privacy concerns. The remaining data are available in the article and supplementary files. Source data are provided with this paper.

## Source data

Source Data

## Code availability

The code used to develop and evaluate the model is available on GitHub with R (version 3.6.2)^39^, 10.5281/zenodo.3991113.
