# Supplementary material for: Machine learning based early warning system enables accurate mortality risk prediction for COVID-19
Source: Nat Commun. 2020 Oct 6;11:5033. doi: 10.1038/s41467-020-18684-2 (PMC7538910; doi:10.1038/s41467-020-18684-2)
Supplement: Supplementary file 1 — Supplementary Information [file 41467_2020_18684_MOESM1_ESM.pdf]

Supplementary Information

"Machine learning based early warning system enables accurate mortality risk prediction for COVID-19"

Gao et. Al

Supplementary Figures

Supplementary Fig. 1. Schematic overview of MRPMC model.

Supplementary Fig. 2. Visualization of feature filtering.

Supplementary Fig. 3. Visualization of the imputation results on categorical features.

Supplementary Fig. 4. Visualization of the imputation results on continuous features.

Supplementary Fig. 5. Multivariable Cox regression analysis of features.

Supplementary Fig. 6. F1 score of MRPMC on training cohort in respect to different probability cutoff value

Supplementary Fig. 7. Calibration curves of MRPMC across cohorts.

Supplementary Fig. 8. Predictive performance of KNN and RF across cohorts.

Supplementary Tables

Supplementary Table 1. Features collected from electronic health records and the corresponding normal range.

Supplementary Table 2. Multivariable cox analysis of features by cohorts.

Supplementary Table 3. Performance of mortality risk prediction of KNN and RF in validation cohorts.

Supplementary Table 4. Relative feature importance in models.

Supplementary Table 5. Differential continuous variables between survivors and non-survivors.

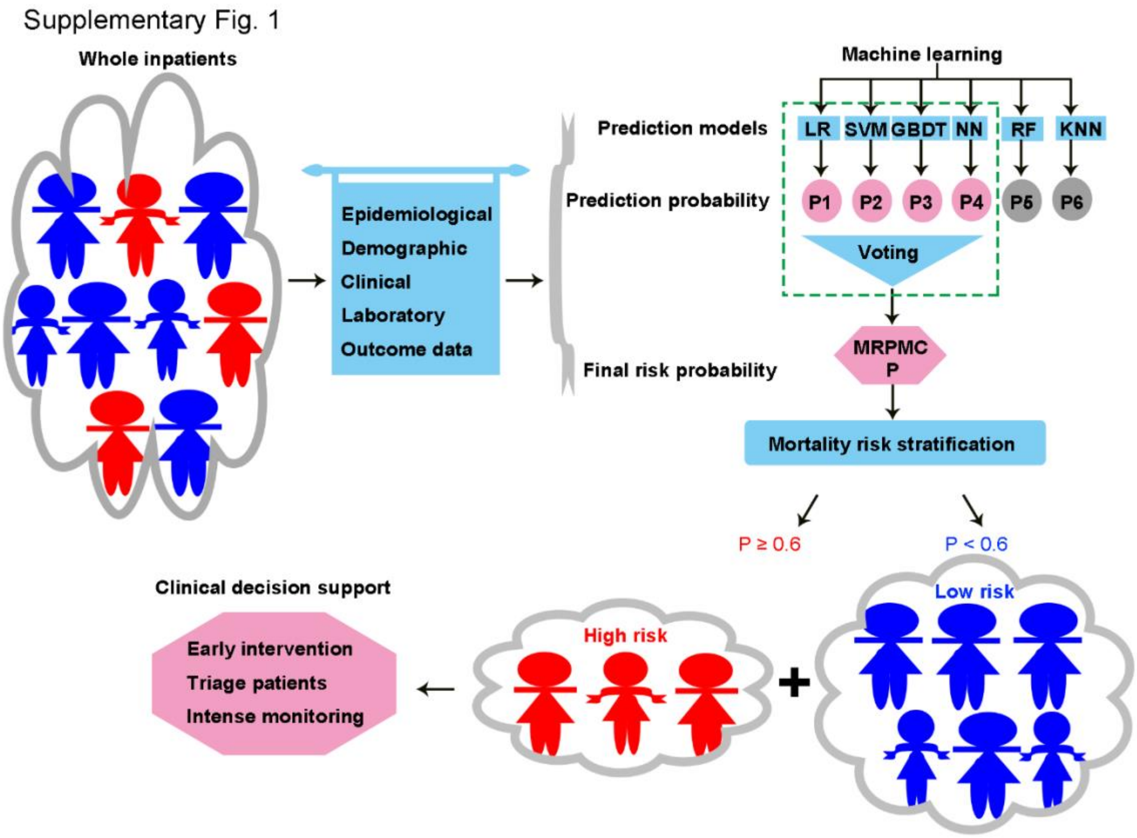

Supplementary Fig. 1 Schematic overview of MRPMC model. With the inpatients' epidemiological, demographic, clinical, and laboratory features as predictors, and the survival outcome as end point, six baseline machine learning models including LR, SVM, GBDT, NN, KNN, and RF were trained. Top four baseline models of best performance (LR, SVM, GBDT, and NN) were selected to construct the ensemble model MRPMC by voting (see Methods). MRPMC finally yielded a morality risk probability ranged from 0 to 1. COVID-19 patients with the probability of less than 0.6 were assigned to be low risk group, otherwise high risk group. *Abbreviations*: MRPMC, mortality risk prediction model for COVID-19; LR, Logistic Regression; SVM, Support Vector Machine; GBDT, Gradient Boosted Decision Tree; NN, Neural Network; KNN, K-Nearest Neighbor; RF, Random Forest; COVID-19, coronavirus disease 2019.

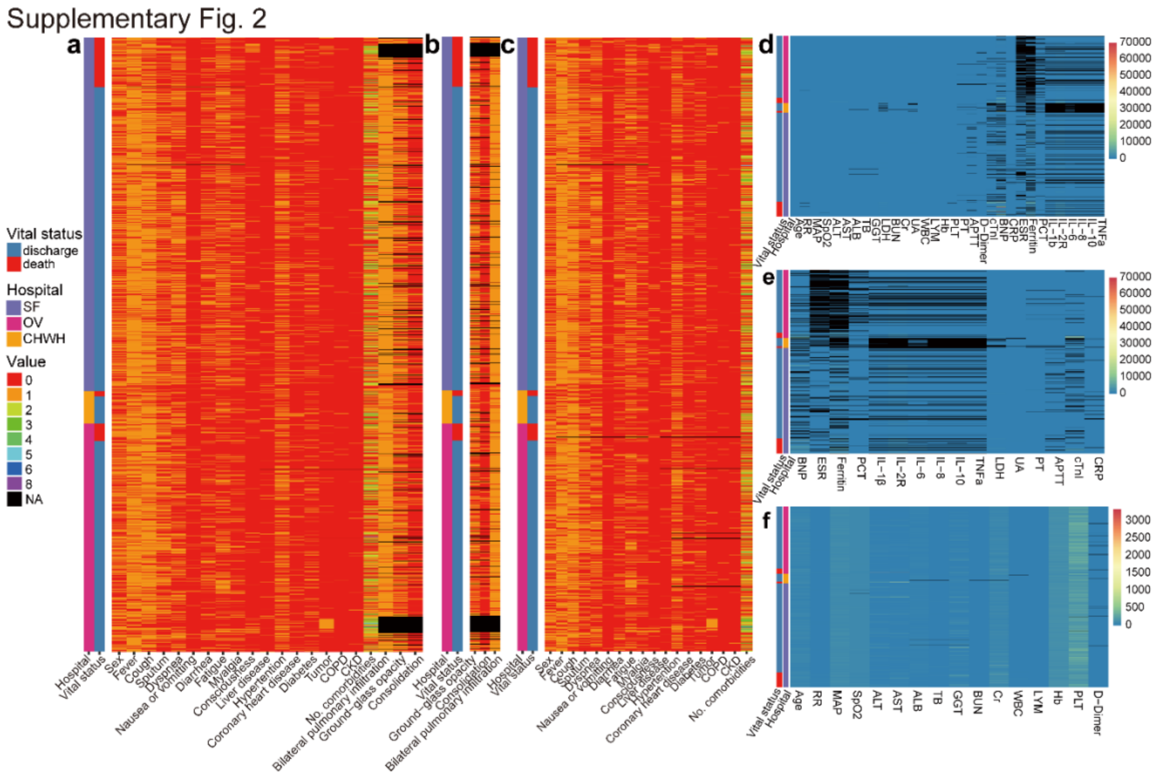

Supplementary Fig. 2 Visualization of feature filtering. a Heatmap of raw data on 21 categorical features. **b** Heatmap of removed three categorical features whose intra-cohort missing rate were larger than or equal to 5%. **c** Heatmap of remained 18 categorical features whose intra-cohort missing rate were less than 5%. **d** Heatmap of raw data on 32 continuous features. **e** Heatmap of removed 16 continuous features whose intra-cohort missing rate were larger than or equal to 5%. **f** Heatmap of remained 16 continuous features whose intra-cohort missing rate were less than 5%. Black tiles refer to missing entries. *Abbreviations*: SF, Sino-French New City Campus of Tongji Hospital. OV, Optical Valley Campus of Tongji Hospital. CHWH, The Central Hospital of Wuhan. COPD, chronic obstructive pulmonary disease. CKD, chronic kidney disease. RR, respiratory rate. MAP, mean arterial pressure. ALT, alanine transaminase. AST, aspartate aminotransferase. ALB, albumin. TB, total bilirubin. GGT, gamma-glutamyl transferase. LDH, lactate dehydrogenase. BUN, blood urea nitrogen. Cr, creatinine. UA, uric acid. WBC, white blood cell count. LYM, lymphocyte count. Hb, hemoglobin. PLT, platelet count. PT, prothrombin time. APTT, activated partial thromboplastin time. BNP, N-terminal B-type natriuretic peptide. CRP, C-reactive protein. ESR, erythrocyte sedimentation rate. PCT, procalcitonin.

Supplementary Fig. 3

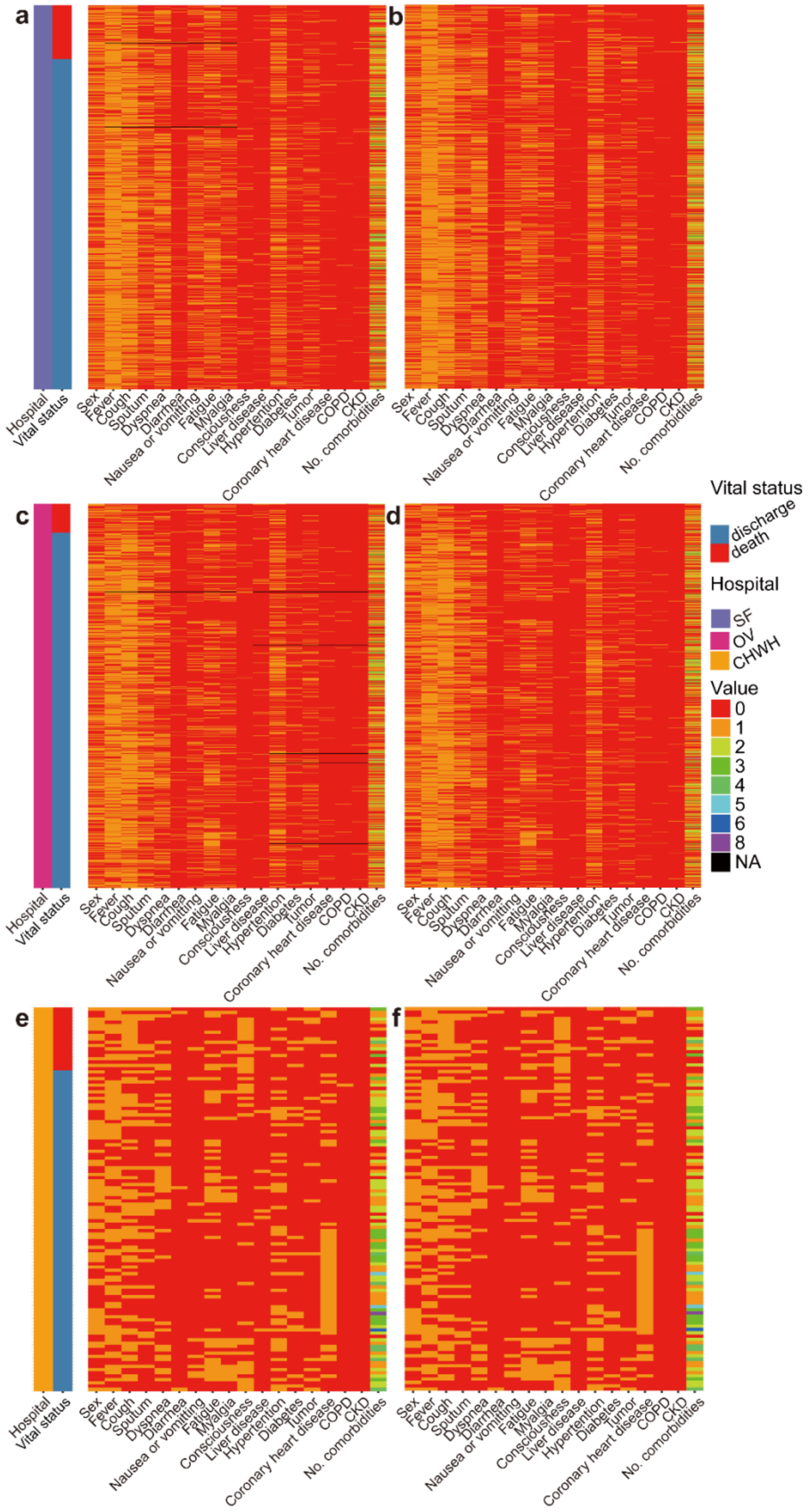

Supplementary Fig. 3 Visualization of the imputation results on categorical features. Heatmap of remained 18 categorical features data before imputation on Sino-French New City Campus of Tongji Hospital (**a)**, Optical Valley Campus of Tongji Hospital (**c)**, and The Central Hospital of Wuhan (**e**), respectively. Heatmap of remained 18 categorical features data after imputation on Sino-French New City Campus of Tongji Hospital (**b)**, Optical Valley Campus of Tongji Hospital (**d)**, and The Central Hospital of Wuhan (**f)**, respectively. Black tiles refer to missing entries. *Abbreviations*: SF, Sino-French New City Campus of Tongji Hospital. OV, Optical Valley Campus of Tongji Hospital. CHWH, The Central Hospital of Wuhan. COPD, chronic obstructive pulmonary disease. CKD, chronic kidney disease.

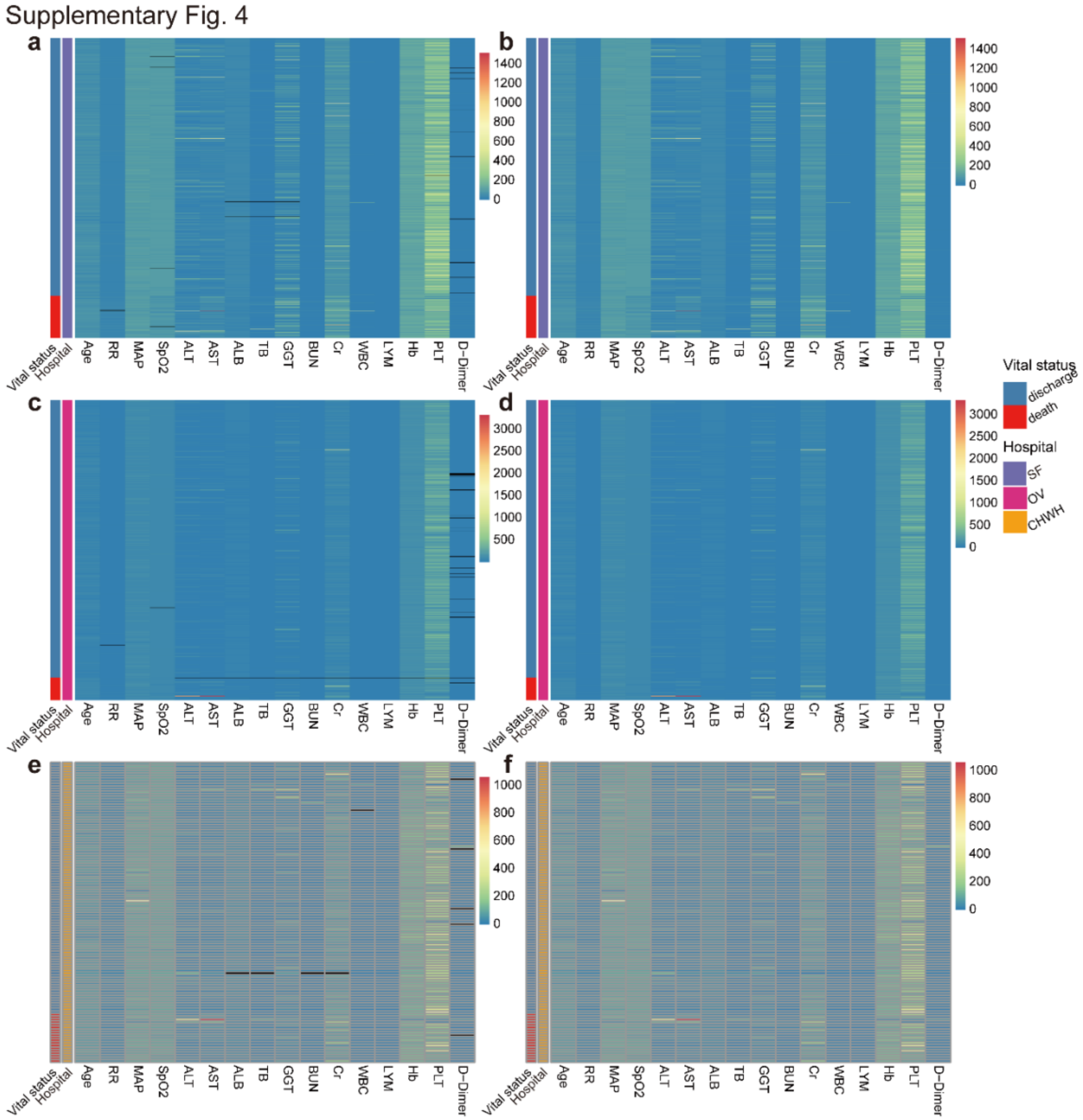

Supplementary Fig. 4 Visualization of the imputation results on continuous features. Heatmap of remained 16 continuous features data before imputation on Sino-French New City Campus of Tongji Hospital (**a**), Optical Valley Campus of Tongji Hospital (**c**), and The Central Hospital of Wuhan (**e**), respectively. Heatmap of remained 16 continuous features data after imputation on Sino-French New City Campus of Tongji Hospital (**b**), Optical Valley Campus of Tongji Hospital (**d**), and The Central Hospital of Wuhan (**f**), respectively. Black tiles refer to missing entries. *Abbreviations*: SF, Sino-French New City Campus of Tongji Hospital. OV, Optical Valley Campus of Tongji Hospital. CHWH, The Central Hospital of Wuhan. RR, respiratory rate. MAP, mean arterial pressure. ALT, alanine transaminase. AST, aspartate aminotransferase. ALB, albumin. TB, total bilirubin. GGT, gamma-glutamyl transferase. BUN, blood urea nitrogen. Cr, creatinine. WBC, white blood cell count. LYM, lymphocyte count. Hb, hemoglobin. PLT, platelet count.

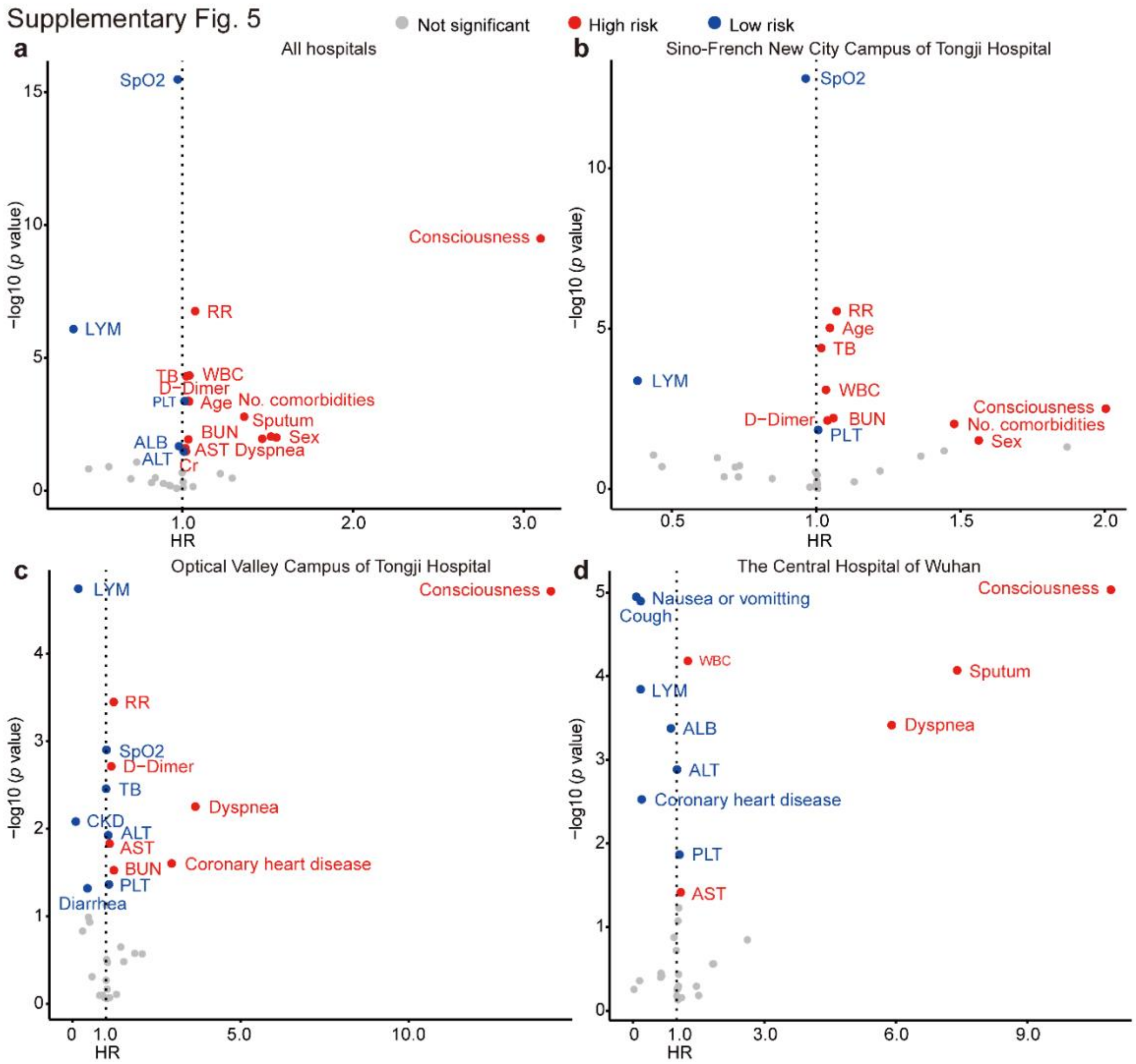

Supplementary Fig. 5 Multivariable Cox regression analysis of features. Hazard Ratio (HR) of 34 features whose intra-cohort missing rate were less than 5% in (**a**) all three hospitals, (**b**) Sino-French New City Campus of Tongji Hospital, (**c**) Optical Valley Campus of Tongji Hospital, and (**d**) The Central Hospital of Wuhan, respectively. Overall survival (OS) was set as the end point. Significant HR < 1 results were colored in blue, significant HR > 1 results were colored in red. The one-side ward test was conducted, $p < 0.05$ was considered as statistically significant. The exact p value of each feature is listed in Supplementary Table 2. *Abbreviations*: COPD, chronic obstructive pulmonary disease. CKD, chronic kidney disease. RR, respiratory rate. MAP, mean arterial pressure. ALT, alanine transaminase. AST, aspartate aminotransferase. ALB, albumin. TB, total bilirubin. GGT, gamma-glutamyl transferase. BUN, blood urea nitrogen. Cr, creatinine. WBC, white blood cell count. LYM, lymphocyte count. Hb, hemoglobin. PLT, platelet count.

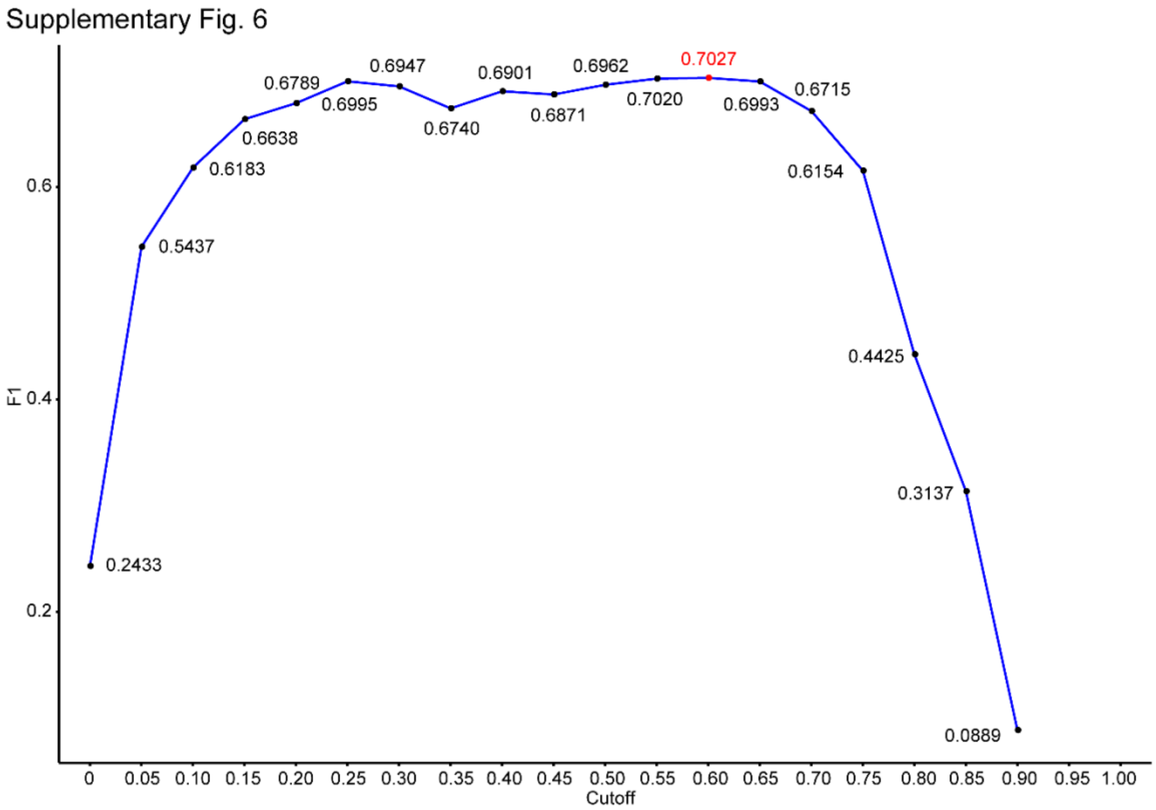

Supplementary Fig. 6 F1 score of MRPMC on training cohort in respect to different probability cutoff value.

Each dot point indicates the corresponding F1 score of the cutoff *p* value. Dot colored with red indicates the highest F1 score (0.7027) with cutoff *p* value of 0.6.

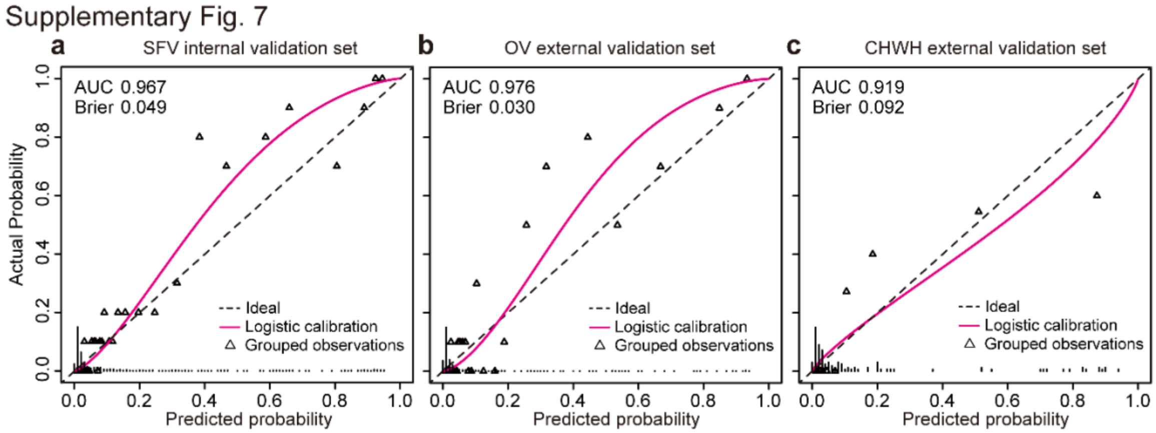

Supplementary Fig. 7 Calibration curves of MRPMC across cohorts. Calibration curves of MRPMC on (**a**) SFV cohort, (**b**) OV cohort, and (**c**) CHWH cohort, respectively. The triangle refers to the observation group. Each group contained an average of 20 observations. The dashed line is the ideal calibration curve. The bottom vertical lines signify the predicted probability distribution. Red curve is the fitted linear logistic calibration curve. *Abbreviations*: AUC, area under the receiver operating characteristics curve. Brier, Brier score. MRPMC, mortality risk prediction model for COVID-19. SFV cohort, internal validation cohort of Sino-French New City Campus of Tongji Hospital. OV cohort, external validation cohort of Optical Valley Campus of Tongji Hospital. CHWH cohort, external validation cohort of The Central Hospital of Wuhan.

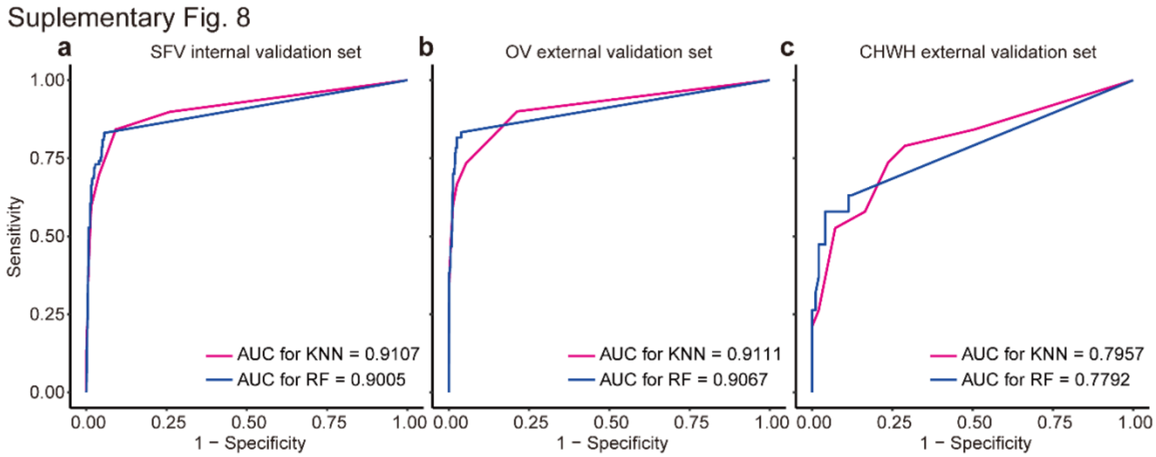

Supplementary Fig. 8 Predictive performance of KNN and RF. Assess the performance of mortality risk prediction of KNN and RF in (**a**) SFV cohort, (**b**) OV cohort, and (**c**) CHWH cohort, respectively. *Abbreviations*: AUC, area under the receiver operating characteristics curve. SFV cohort, internal validation cohort of Sino-French New City Campus of Tongji Hospital. OV cohort, external validation cohort of Optical Valley Campus of Tongji Hospital. CHWH cohort, external validation cohort of The Central Hospital of Wuhan. KNN, K-Nearest Neighbor. RF, Random Forest.

Supplementary Table 1. Features collected and the corresponding normal range.

Features	Normal range
Lactate dehydrogenase, U per L (LDH)	Female, LDH＜214; Male, LDH＜225
SpO2, %	＞93%
Procalcitonin, ng per mL (PCT)	＜0.25
Blood urea nitrogen, umol per L (BUN)	Female, BUN＜8.8; Male, BUN＜9.5
Respiratory rate, per min (RR)	≤24
N-terminal B-type natriuretic peptide (NT-proBNP)	＜247
High-sensitivity cardiac troponin I, pg per mL (cTnI)	≤34.2
White blood cell count, ×10^9 per L (WBC)	3.5≤WBC＜9.5
D-dimer, ug per mL (D-Dimer)	＜0.5
Ground-glass opacity	No
Lymphocyte count, ×10^9 per L (LYM)	1.10≤LYM＜3.20
Prothrombin time, s (PT)	＜14.5
C-reactive protein, mg per L (CRP)	＜1
Consolidation	No
Interleukin-6, pg per mL (IL-6)	＜7
Interleukin-8, pg per mL (IL-8)	＜62
Interleukin-2R, U per mL (IL-2R)	≤710
Albumin, g per L (ALB)	＞35
Age, years	Not Applicable
Ferritin, ug per L	Female, Ferritin≤150; Male, Ferritin≤400
Interleukin-10, pg per mL (IL-10)	＜9.1
Creatinine, umol per L (Cr)	＜104
Platelet count, ×10^9 per L (PLT)	125≤PLT＜350
Total bilirubin, umol per L (TB)	＜20
Activated partial thromboplastin time, s (APTT)	＜42
Uric acid, umol per L (UA)	Female.UA＜339.2; Male.UA＜416.5
Tumor Necrosis Factor Alpha (TNFα)	＜8.1
Gamma-Glutamyl Transferase, U per L (GGT)	Female, GGT≤42; Male, GGT≤71
Mean arterial pressure, mmHg (MAP)	≥70
Alanine transaminase, U per L (ALT)	Female, ALT≤33; Male, ALT≤41
Erythrocyte sedimentation rate, mm per h (ESR)	≤20
Aspartate aminotransferase, U per L (AST)	Female, AST≤32; Male, AST≤40
Number of comorbidities	Not Applicable
Hemoglobin, g per L (Hb)	Female, Hb≥115; Male, Hb≥130
Dyspnea	Not Applicable
Interleukin-β (IL-1β)	＜5
Fatigue	Not Applicable
Consciousness	Not Applicable
Sputum	Not Applicable
Myalgia	Not Applicable

Diabetes	Not Applicable
Sex	Not Applicable
Hypertension	Not Applicable
Fever	Not Applicable
Diarrhea	Not Applicable
Chronic liver disease (CLD)	Not Applicable
Vomiting	Not Applicable
Bilateral pulmonary infiltration	Not Applicable
Cough	Not Applicable
Chronic kidney disease (CKD)	Not Applicable
Chronic obstructive pulmonary disease (COPD)	Not Applicable
Coronary heart disease (CHD)	Not Applicable
Tumor	Not Applicable

Supplementary Table 2. Multivariable cox analysis of features by cohorts.

Features	All hospitals		SF		OV		CHWH	
	HR (95% CI)	*p* value	HR (95% CI)	*p* value	HR (95% CI)	*p* value	HR (95% CI)	*p* value
WBC	1.02 (1.01–1.04)	5e–05	1.02 (1.01–1.04)	9e–04	0.99 (0.87–1.12)	0.88	1.19 (1.09–1.29)	7e–05
Tumor	1.29 (0.76–2.17)	0.34	1.87 (1.00–3.48)	0.05	1.28 (0.21–7.66)	0.79	0.63 (0.23–1.77)	0.39
TB	1.01 (1.00–1.01)	6e–05	1.01 (1.00–1.01)	5e–05	0.91 (0.86–0.97)	0.004	0.98 (0.94–1.04)	0.56
Sputum	1.50 (1.10–2.05)	0.01	1.44 (0.98–2.13)	0.07	1.08 (0.49–2.38)	0.86	7.34 (2.71–19.89)	9e–05
SpO2	0.96 (0.95–0.97)	<0.0001	0.95 (0.94–0.97)	<0.0001	0.93 (0.88–0.97)	0.001	0.97 (0.92–1.02)	0.19
RR	1.06 (1.04–1.08)	<0.0001	1.06 (1.04–1.09)	<0.0001	1.14 (1.06–1.23)	4e–04	0.92 (0.82–1.03)	0.13
PLT	0.997 (0.995–0.999)	5e–04	0.997 (0.995–1.00)	0.02	0.996 (0.991–1.00)	0.05	0.99 (0.99–1.00)	0.01
Myalgia	0.96 (0.65–1.42)	0.84	1.13 (0.70–1.81)	0.62	1.83 (0.63–5.29)	0.27	0.61 (0.19–1.94)	0.4
MAP	0.99 (0.98–1.00)	0.22	1.00 (0.98–1.01)	0.74	0.97 (0.95–1.00)	0.07	1.01 (0.97–1.04)	0.74
LYM	0.35 (0.23–0.53)	<0.0001	0.37 (0.21–0.65)	5e–04	0.09 (0.03–0.27)	2e–05	0.11 (0.03–0.34)	2e–04
Hypertension	0.92 (0.64–1.34)	0.68	0.65 (0.39–1.10)	0.11	2.05 (0.57–7.33)	0.27	1.43 (0.49–4.16)	0.51
Hb	0.999 (0.991–1.01)	0.79	1.00 (0.99–1.00)	0.3	1.01 (0.99–1.04)	0.34	1.03 (1.00–1.05)	0.06
GGT	0.999 (0.997–1.00)	0.49	0.999 (0.997–1.00)	0.59	1.002 (0.99–1.01)	0.68	1.01 (0.99–1.02)	0.55
Sex	1.53 (1.10–2.14)	0.01	1.55 (1.04–2.31)	0.03	1.50 (0.66–3.40)	0.33	2.59 (0.72–9.28)	0.14
Fever	0.73 (0.51–1.05)	0.09	0.73 (0.46–1.17)	0.19	0.48 (0.20–1.20)	0.12	1.80 (0.62–5.20)	0.28
Fatigue	1.06 (0.78–1.44)	0.73	0.98 (0.66–1.44)	0.9	0.93 (0.41–2.1)	0.85	0.62 (0.22–1.73)	0.36
Dyspnea	1.45 (1.08–1.95)	0.01	1.22 (0.85–1.74)	0.28	3.57 (1.45–8.82)	0.006	5.84 (2.2–15.53)	4e–04
Diarrhea	1.22 (0.88–1.68)	0.24	1.36 (0.95–1.96)	0.1	0.36 (0.13–1.00)	0.05	1.48 (0.26–8.58)	0.66
Diabetes	0.84 (0.58–1.20)	0.33	0.84 (0.53–1.36)	0.49	0.45 (0.17–1.18)	0.1	1.82 (0.62–5.33)	0.28
Cr	1.002 (1.000–1.003)	0.03	1.00 (0.999–1.00)	0.38	1.00 (0.99–1.01)	0.88	1.01 (1.00–1.02)	0.09
Cough	0.92 (0.63–1.33)	0.65	1.00 (0.65–1.55)	0.99	0.86 (0.27–2.78)	0.8	0.11 (0.04–0.30)	1e–05
COPD	0.69 (0.31–1.54)	0.37	0.68 (0.26–1.77)	0.43	0.27 (0.05–1.59)	0.15	6e–04(1.06e–14–3.79e+07)	0.56
Consciousness	3.08 (2.17–4.39)	<0.0001	1.99 (1.25–3.17)	0.004	14.15 (4.19–47.86)	2e–05	10.85 (3.77–31.20)	<0.00001
CKD	0.45 (0.15–1.36)	0.16	0.46 (0.14–1.53)	0.21	0.01 (5e–04–0.34)	0.009	1(1–1)	nonsignificant
BUN	1.02 (1.00–1.03)	0.01	1.05 (1.01–1.09)	0.007	1.14 (1.01–1.29)	0.03	1.02 (0.97–1.06)	0.51
AST	1.01 (1.00–1.01)	0.04	0.999 (0.993–1.01)	0.78	1.02 (1.00–1.03)	0.02	1.03 (1.00–1.05)	0.04
ALT	0.99 (0.99–1.00)	0.04	1.00 (0.99–1.01)	0.71	0.98 (0.96–0.99)	0.01	0.94 (0.90–0.98)	0.001
ALB	0.96 (0.93–1.00)	0.02	0.999 (0.96–1.04)	0.94	0.97 (0.89–1.06)	0.54	0.80 (0.71–0.91)	4e–04
Age	1.02 (1.01–1.04)	5e–04	1.04 (1.02–1.06)	1e–05	0.98 (0.96–1.01)	0.31	1.02 (0.98–1.07)	0.37
No. comorbidities	1.35 (1.12–1.62)	0.002	1.47 (1.09–1.97)	0.01	1.41 (0.81–2.45)	0.23	1.09 (0.71–1.67)	0.7
Nausea or vomiting	0.81 (0.44–1.51)	0.52	0.73 (0.33–1.60)	0.43	0.56 (0.1–2.98)	0.49	0.01 (0.001–0.08)	1e–05
Liver disease	0.56 (0.27–1.18)	0.13	0.43 (0.17–1.14)	0.09	0.78 (0.11–5.56)	0.81	0.13 (7e–04–23.42)	0.44
D-Dimer	1.02 (1.01–1.03)	5e–04	1.03 (1.01–1.05)	0.008	1.07 (1.02–1.12)	0.002	0.98 (0.89–1.08)	0.67
CHD	0.88 (0.59–1.33)	0.55	0.72 (0.42–1.21)	0.21	2.87 (1.13–7.25)	0.03	0.13 (0.04–0.51)	0.003

The one-side ward test was conducted, and $p < 0.05$ was considered as statistically significant. *Abbreviations*: WBC, white blood cell count. TB, total bilirubin. RR, respiratory rate. PLT, platelet count. MAP, mean arterial pressure. LYM, lymphocyte count. Hb, hemoglobin. GGT, gamma-glutamyl transferase. Cr, creatinine. COPD, chronic obstructive pulmonary disease. CKD, chronic kidney disease. BUN, blood urea nitrogen. AST, aspartate aminotransferase. ALT, alanine transaminase. ALB, albumin. No. comorbidities, number of comorbidities. CHD, coronary heart disease. HR, hazard ratio. SF, Sino-French New City Campus of Tongji Hospital. OV, Optical Valley Campus of Tongji Hospital. CHWH, The Central Hospital of Wuhan.

Supplementary Table 3. Relative feature importance in models.

Feature	MRPMC	SVM	GBDT	LR	RF	KNN	NN
Age	65.91	75.01	24.16	68.99	54.76	75.01	67.82
RR	67.78	100.39	28.91	96.37	89.20	100.39	30.52
SpO2	72.38	103.14	53.85	110.00	108.49	103.14	24.43
ALB	75.11	89.42	25.07	78.50	60.38	89.42	74.72
BUN	71.50	110.00	110.00	96.01	110.00	110.00	10.00
Lymphocyte	70.67	93.46	44.24	83.05	73.63	93.46	49.84
Platelet	64.17	56.67	17.61	49.15	56.20	56.67	94.64
D-Dimer	105.33	107.00	99.18	99.24	85.00	107.00	110.00
Sex	54.30	45.31	10.51	47.03	19.18	45.31	79.71
Fever	48.90	25.06	10.00	26.74	15.78	25.06	96.28
Sputum	44.23	22.69	10.97	29.24	17.06	22.69	82.90
Consciousness	40.83	39.53	10.00	37.70	30.49	39.53	53.00
CKD	40.01	10.00	10.00	10.00	10.00	10.00	95.76
No. comorbidities	45.62	53.22	13.76	49.13	28.18	53.22	45.71

Abbreviations: MRPMC, mortality risk prediction model for COVID-19. SVM, Support Vector Machine. GBDT, Gradient Boosted Decision Tree. LR, Logistic Regression. RF, Random Forest. KNN, K-Nearest Neighbor. NN, Neural Network. RR, respiratory rate. ALB, albumin. BUN, blood urea nitrogen. CKD, chronic kidney disease. No. comorbidities, number of comorbidities.

Supplementary Table 4. Differential continuous variables between survivors and non-survivors.

Features	Death (*n* = 254)	Discharge (*n* =1906)	*p* value	test
BUN (median [IQR])	8.2 [5.8, 12.9]	4.2 [3.3, 5.3]	<0.001	Two-sided Wilcoxon test
D-Dimer (median [IQR])	4.16 [1.42, 21.00]	0.61 [0.32, 1.35]	<0.001	Two-sided Wilcoxon test
RR (median [IQR])	28 [22, 33]	20 [20, 22]	<0.001	Two-sided Wilcoxon test
LYM (median [IQR])	0.59 [0.43, 0.83]	1.22 [0.85, 1.62]	<0.001	Two-sided Wilcoxon test
ALB (median [IQR])	30.8 [28.0, 34.0]	36.35 [32.8, 40.1]	<0.001	Two-sided Wilcoxon test
PLT (median [IQR])	159.0 [108.5, 222.5]	225.0 [172.0, 295.3]	<0.001	Two-sided Wilcoxon test
Age (median [IQR])	70 [63, 77]	61 [49, 69]	<0.001	Two-sided Wilcoxon test
SpO2 (median [IQR])	85 [75, 92]	96 [93, 97]	<0.001	Two-sided Wilcoxon test

Abbreviations: BUN, blood urea nitrogen. RR, respiratory rate. LYM, lymphocyte count. ALB, albumin. PLT, platelet count. SpO2, oxygen saturation. IQR, interquartile ranges.

Supplementary Table 5. Performance metrics for mortality risk prediction of KNN and RF in cohorts.

	AUC (95% CI)	Accuracy (95% CI)	Sensitivity (95% CI)	Specificity (95% CI)	PPV (95% CI)	NPV (95% CI)	F1	Kappa	Brier
Internal validation cohort (SFV)									
KNN	0.9058 (0.8677–0.9440)	92.8% (90.4%–94.7%)	53.9% (43.0%–64.6%)	99.3% (98.1%–99.8%)	92.3% (81.5%–97.9%)	92.8% (90.4%–94.8%)	0.681	0.643	0.060
RF	0.9094 (0.8702–0.9486)	93.7% (91.5%–95.5%)	71.9% (61.4%–80.9%)	97.4% (95.6%–98.6%)	82.1% (71.7%–89.8%)	95.4% (93.3%–97.0%)	0.767	0.730	0.062
External validation cohort (OV)									
KNN	0.8974 (0.8482–0.9466)	94.5% (92.7%–96.0%)	35.0% (23.1%–48.4%)	99.3% (98.4%–99.8%)	80.8% (60.7%–93.5%)	95.0% (93.2%–96.4%)	0.489	0.464	0.040
RF	0.9143 (0.8674–0.9612)	96.5% (95.0%–97.7%)	68.3% (55.0%–79.7%)	98.8% (97.7%–99.4%)	82.0% (68.6%–91.4%)	97.5% (96.1%–98.5%)	0.746	0.727	0.035
External validation cohort (CHWH)									
KNN	0.8247 (0.7199–0.9296)	85.3% (77.6%–91.2%)	52.6% (28.9%–75.6%)	91.6% (84.4%–96.4%)	55.6% (30.8%–78.5%)	90.8% (83.3%–95.7%)	0.541	0.453	0.129
RF	0.7949 (0.6772–0.9126)	88.8% (81.6%–93.9%)	52.6% (28.9%–75.6%)	95.9% (89.8%–98.9%)	71.4% (41.9%–91.6%)	91.2% (83.9%–95.9%)	0.606	0.543	0.112

Abbreviations: SFV, internal validation cohort of Sino-French New City Campus of Tongji Hospital. OV, Optical Valley Campus of Tongji Hospital. CHWH, The Central Hospital of Wuhan. KNN, K-Nearest Neighbor. RF, Random Forest. AUC, area under the receiver operating characteristics curve. PPV, positive predictive value. NPV, negative predictive value. 95% CI, 95% confidence interval. Brier, Brier score.
